# Non-coding RNA in cancer drug resistance: Underlying mechanisms and clinical applications

**DOI:** 10.3389/fonc.2022.951864

**Published:** 2022-08-17

**Authors:** Xuehao Zhou, Xiang Ao, Zhaojun Jia, Yiwen Li, Shouxiang Kuang, Chengcheng Du, Jinyu Zhang, Jianxun Wang, Ying Liu

**Affiliations:** ^1^ School of Basic Medical Sciences, Qingdao Medical College, Qingdao University, Qingdao, China; ^2^ College of New Materials and Chemical Engineering, Beijing Key Laboratory of Enze Biomass Fine Chemicals, Beijing Institute of Petrochemical Technology, Beijing, China; ^3^ Institute for Translational Medicine, The Affiliated Hospital of Qingdao University, Qingdao Medical College, Qingdao University, Qingdao, China

**Keywords:** non-coding RNA, cancer, drug resistance, biomarker, therapeutic target

## Abstract

Cancer is one of the most frequently diagnosed malignant diseases worldwide, posing a serious, long-term threat to patients’ health and life. Systemic chemotherapy remains the first-line therapeutic approach for recurrent or metastatic cancer patients after surgery, with the potential to effectively extend patient survival. However, the development of drug resistance seriously limits the clinical efficiency of chemotherapy and ultimately results in treatment failure and patient death. A large number of studies have shown that non-coding RNAs (ncRNAs), particularly microRNAs, long non-coding RNAs, and circular RNAs, are widely involved in the regulation of cancer drug resistance. Their dysregulation contributes to the development of cancer drug resistance by modulating the expression of specific target genes involved in cellular apoptosis, autophagy, drug efflux, epithelial-to-mesenchymal transition (EMT), and cancer stem cells (CSCs). Moreover, some ncRNAs also possess great potential as efficient, specific biomarkers in diagnosis and prognosis as well as therapeutic targets in cancer patients. In this review, we summarize the recent findings on the emerging role and underlying mechanisms of ncRNAs involved in cancer drug resistance and focus on their clinical applications as biomarkers and therapeutic targets in cancer treatment. This information will be of great benefit to early diagnosis and prognostic assessments of cancer as well as the development of ncRNA-based therapeutic strategies for cancer patients.

## Introduction

Cancer is the second leading cause of death after cardiovascular disease globally, representing a serious threat to patients’ life and health (1, 2). Based on recent statistics from the International Agency for Research, approximately 19.3 million new cancer cases and more than 10.0 million deaths occurred in 2020 (3). Currently, surgical resection, radiation, endocrine therapy, targeted therapy, and systemic chemotherapy are the main methods of cancer treatment. Among them, systemic chemotherapy is the most effective therapeutic option for all stages of cancer, with the potential to improve patients’ prognosis in the short term (4–6). It has been reported that chemotherapy could extend the overall survival (OS) of patients with advanced cancer by 6.7 months compared to patients only treated with best supportive care (7). However, the emergency of drug resistance significantly limits the clinical application of chemotherapeutic agents, ultimately resulting in treatment failure and patient death. Drug resistance has become an immense obstacle in cancer treatment (8). The underlying mechanisms involved in drug resistance are considerably complex and have not been fully elucidated. Therefore, a better understanding of the mechanisms responsible for drug resistance will provide opportunities for the development of precise therapeutic strategies for cancer patients.

Non-coding RNAs (ncRNAs), such as microRNAs (miRNAs), long non-coding RNAs (lncRNAs), and circular RNAs (circRNAs), are a large group of transcripts that have no protein coding potential. They were recognized as by-products of transcription without biological function in the past long period of time (9). In recent years, an increasing amount of evidence has suggested that ncRNAs are crucial regulators in almost all cellular processes, such as transcription, apoptosis, proliferation, and differentiation (10, 11). They play crucial roles in the regulation of a variety of physiological and pathological processes. The dysregulation of ncRNAs has been shown to be closely associated with a variety of diseases, particularly cancer (12–14). For instance, the overexpression of miRNA-200a-3p was found to significantly facilitate cell proliferation, migration, and invasion as well as induce apoptosis in gastric cancer (GC) by directly targeting DLC-1 (15). LncRNA ITGB8-AS1 was found to promote cell proliferation, colony formation, and tumor growth in colorectal cancer (CRC) by upregulating ITGA3 and ITGB3 *via* sponging miR-33b-5p and let-7c-5p/let-7d-5p (16). Furthermore, circRNA C190 overexpression facilitated the proliferation, and migration of non-small cell lung carcinoma (NSCLC) cell lines by targeting CDK1 and CDK6 *via* sequestrating miR-142-5p (17). Notably, ncRNA dysregulation contributes to the development of cancer drug resistance *via* various mechanisms, such as the inhibition of apoptosis, enhancement of epithelial-to-mesenchymal transition (EMT), and induction of autophagy (18–20). In addition, the differential expression patterns of ncRNAs endow them with great potential as biomarkers and therapeutic targets for cancer patients.

In this review, we summarize the recent findings on the regulatory mechanisms of ncRNAs in cancer drug resistance and highlight their clinical applications as promising biomarkers and therapeutic targets for cancer patients. A better understanding of the underlying mechanisms of ncRNAs in drug resistance may offer an opportunity to develop ncRNA-based therapeutic strategies for cancer patients against drug resistance.

## Overview of ncRNAs

### Classification of ncRNAs

It has been reported that ncRNAs make up about 98% of the human genome (21). With the continuous development of high-throughput sequencing technologies, an increasing number of ncRNAs are being identified in eukaryotic cells. According to distinguished classification standards, ncRNAs can be divided into a variety of categories. For instance, ncRNAs are classified into housekeeping ncRNAs (e.g., rRNAs and tRNAs) and regulatory ncRNAs (e.g., miRNAs, circRNAs and lncRNAs) based on their cellular functions. According to their transcript size, ncRNAs are divided into lncRNAs (> 200 nucleotides) and small ncRNAs (< 200 nucleotides), including miRNAs, small interfering RNAs (siRNAs), and piwi-interacting RNAs (piRNAs) (22, 23). Besides, lncRNAs are sorted into two categories, linear lncRNAs and circular lncRNAs based on their structure (24). Moreover, according to the role of lncRNAs in gene expression regulation, they are classified as cis-lncRNAs or trans-lncRNAs (25). In addition, ncRNAs can also be divided into distinct categories based on their subcellular localization (e.g., small nuclear RNAs and cytoplasm-located siRNAs) and genomic origins (including sense or antisense ncRNAs, bidirectional ncRNAs, intronic ncRNAs, and intergenic ncRNAs) (26). Collectively, scientific and systematic classification will be of great benefit in better understanding the characteristics of ncRNAs.

### Biogenesis of ncRNAs

**Figure 1:**
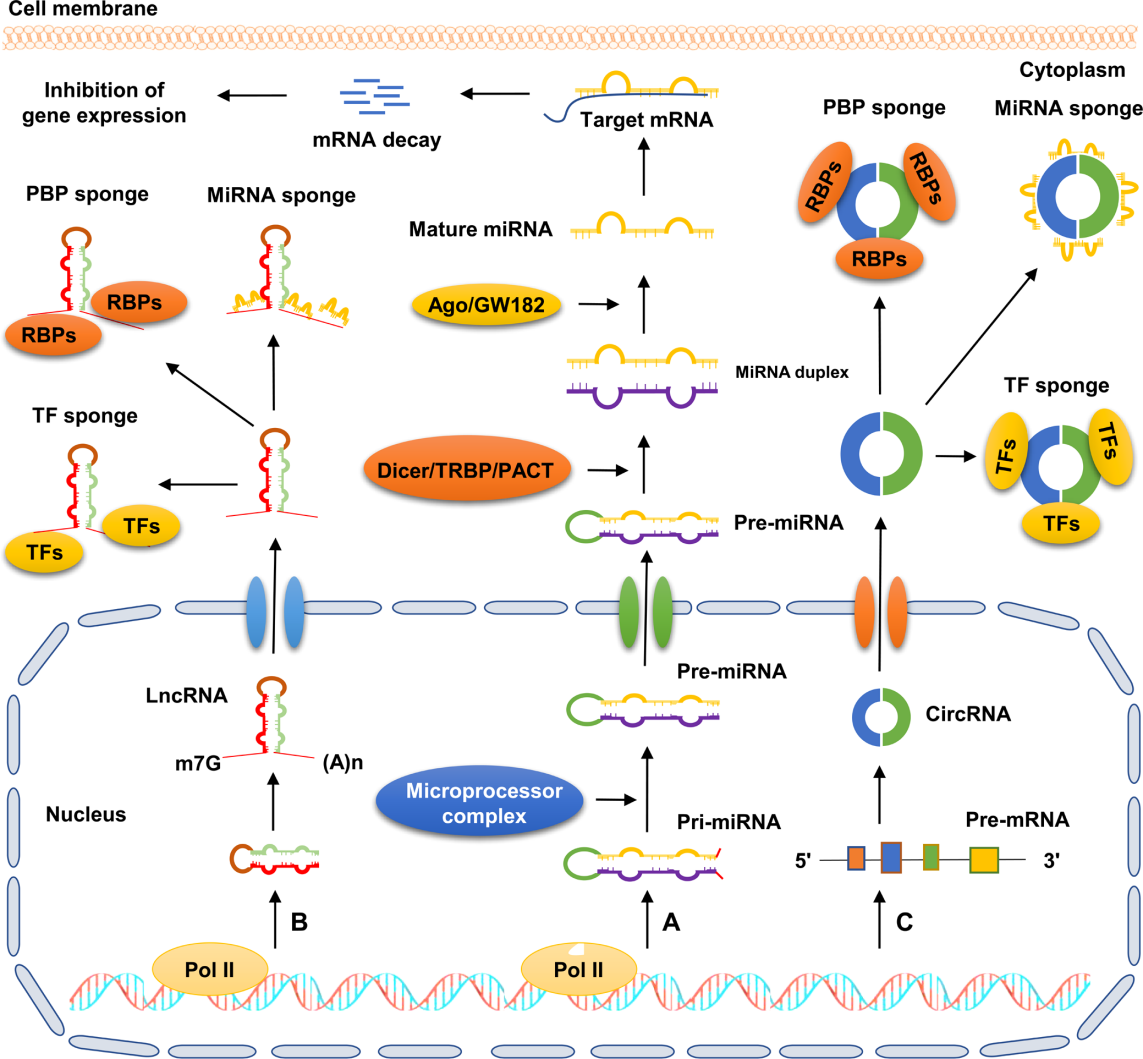
Schematic diagram of ncRNA biogenesis and action patterns. **(A)** Pri-miRNA is transcribed by RNA polymerasel II from genomic loci and further processed into pre-miRNA by microprocessor complex. Subsequently, pre-miRNA is exported to the cytoplasm and further processed into double-stranded miRNA *via* the Dicer/TRBP/PACT complex. Next, with the help of Ago/GW182, the double-stranded miRNA is processed into mature miRNA, which directly binds to the 3’-UTR of target mRNA, and then facilitates its degradation. **(B)** LncRNA transcribed by RNA polymerase II is exported to the cytoplasm. Subsequently, lncRNA exerts its biological role by acting as sponges of miRNAs, RBPs, and TFs. **(C)** CircRNA is mainly derived from precursor mRNAs *via* back-splicing reaction, by which the single strand of circRNA forms a covalently closed-loop structure. CircRNA plays crucial roles in cellular processes by serving as sponges of miRNAs, RBPs, and TFs.

The mechanisms of ncRNA biogenesis are extremely complicated, and individual ncRNA categories possess unique characteristics (**Figure 1**). For instance, both miRNAs and lncRNAs are transcribed by RNA polymerase II (Pol II) from genomic loci. Primary miRNAs (pri-miRNAs) are subsequently catalyzed by a microprocessor complex consisting of DiGeorge syndrome critical region 8 (DGCR8) and Drosha to generate precursor miRNAs (pre-miRNAs). Pre-miRNAs are translocated from the nucleus to the cytoplasm, and then processed into double-stranded miRNAs by the Dicer/TRBP/PACT complex. Finally, the double-stranded miRNAs are processed into mature miRNAs by a series of regulators, including helicase and the RNA-induced silencing complex (RISC) (27). Different from miRNAs, lncRNAs contain 5’ caps and 3’ poly(A) tails. Most lncRNAs undergo a canonical mechanism similar to the biogenesis of mRNAs, by which they are often capped by 7-methyl guanosine at the 5’ end of Pol II transcripts, polyadenylated at their 3′ ends, and spliced similarly to mRNAs (28). CircRNAs are a novel type of ncRNAs characterized by the formation of covalently closed-loop structures without 5’ caps and 3’ tails. CircRNAs are mainly produced from precursor mRNAs *via* a unique mechanism called back-splicing reaction, in which a downstream splice donor site binds to an upstream splice acceptor site to form a single-strand, covalently closed-loop structure (29).

The biogenesis of ncRNAs is widely regulated by various factors, such as trans-acting factors, RNA binding proteins (RBPs), and epigenetic modifications. For instance, the overexpression of poly(A)-binding protein nuclear 1 (PABPN1) was found to facilitate the turnover of non-coding transcripts *via* a polyadenylation-dependent mechanism, indicating its negative role in modulating the processing of certain ncRNAs (30). Alternative splicing factor1/pre-mRNA splicing factor SF2 (ASF/SF2) is a classical RBP encoded by the *SFRS1* gene. Wu et al. showed that SF2/ASF overexpression facilitated the maturation process of a series of miRNAs, including miR-7, miR-29b, miR-221, and miR-222. Consistent with this, the knockdown of SF2/ASF resulted in a decreased level of mature miR-7 (31). N^6^-methyladenosine (m^6^A) is a well-studied RNA modification that plays crucial roles in distinct processes modulating RNA metabolism, such as the splicing, stability, and translation of mRNA (32). Timoteo et al. revealed that specific m6As promoted circRNA back-splicing reaction in a METTL3- and YTHDC1-dependent manner, whereas the mutation of the m6A sites significantly decreased the circRNA levels, which was paralleled by a strong increase in the precursor RNA (33). Although some progress has been made in recent years, ncRNA biogenesis and its regulatory mechanisms are still not fully understood. Continuous in-depth studies will be beneficial not only in differentiating ncRNAs from protein-coding RNAs but also in deciphering their functional significance.

### Patterns of ncRNA action

A large amount of evidence suggests that ncRNAs are involved in almost all physiological and pathological processes, including tissue development, cancer progression, and drug resistance. They play crucial roles in these processes *via* distinct molecular mechanisms, such as regulating the expression of specific target genes, altering the function and activity of proteins, and targeting related signaling pathways (34–36). All these mechanisms are mainly based on the interaction of ncRNAs with DNA, RNA, and proteins (**Figure 1**). For instance, miRNAs are 19–25 nucleotides in length and play crucial roles in pivotal cellular processes by regulating specific gene expression at the post-transcriptional level (37, 38). They inhibit the expression of specific genes by directly binding to 3′ untranslated regions (UTRs) of their target mRNAs. One single miRNA can simultaneously control the expression of multiple target genes involved in distinct cellular processes (e.g., invasion, metastasis, and cell cycle), while one gene can also be regulated by several miRNAs (39). LncRNAs and circRNAs have been shown to exert their biological functions by acting as sponges or molecular sinks for miRNAs, RBPs, and transcription factors to specifically modulate their target gene expression. These ncRNAs are also called intracellular competitive endogenous RNAs (ceRNAs) (40, 41). For instance, lncRNA SLC25A25-AS1 was found to promote proliferation, migration, and invasion, and induced apoptosis in NSCLC A549 and H460 cells by upregulating integrin α2 *via* sponging miR-195-5p (42). Due to their central role in physiological and pathological processes, the dysregulation of ncRNAs is closely associated with the occurrence and development of many diseases including cancer. In fact, the aberrant expression of ncRNAs has been observed in cancer tissues and cell lines. They are involved in the regulation of cancer progression by serving as oncogenes or tumor suppressors (10). In addition, ncRNAs are also crucial regulators in the development of cancer drug resistance (10). In-depth investigations of the underlying mechanism of ncRNAs in cancer drug resistance could contribute to the precise treatment of cancer patients, particularly those with poor response to chemotherapy.

## Mechanisms of ncRNAs in mediating cancer drug resistance

**Figure 2:**
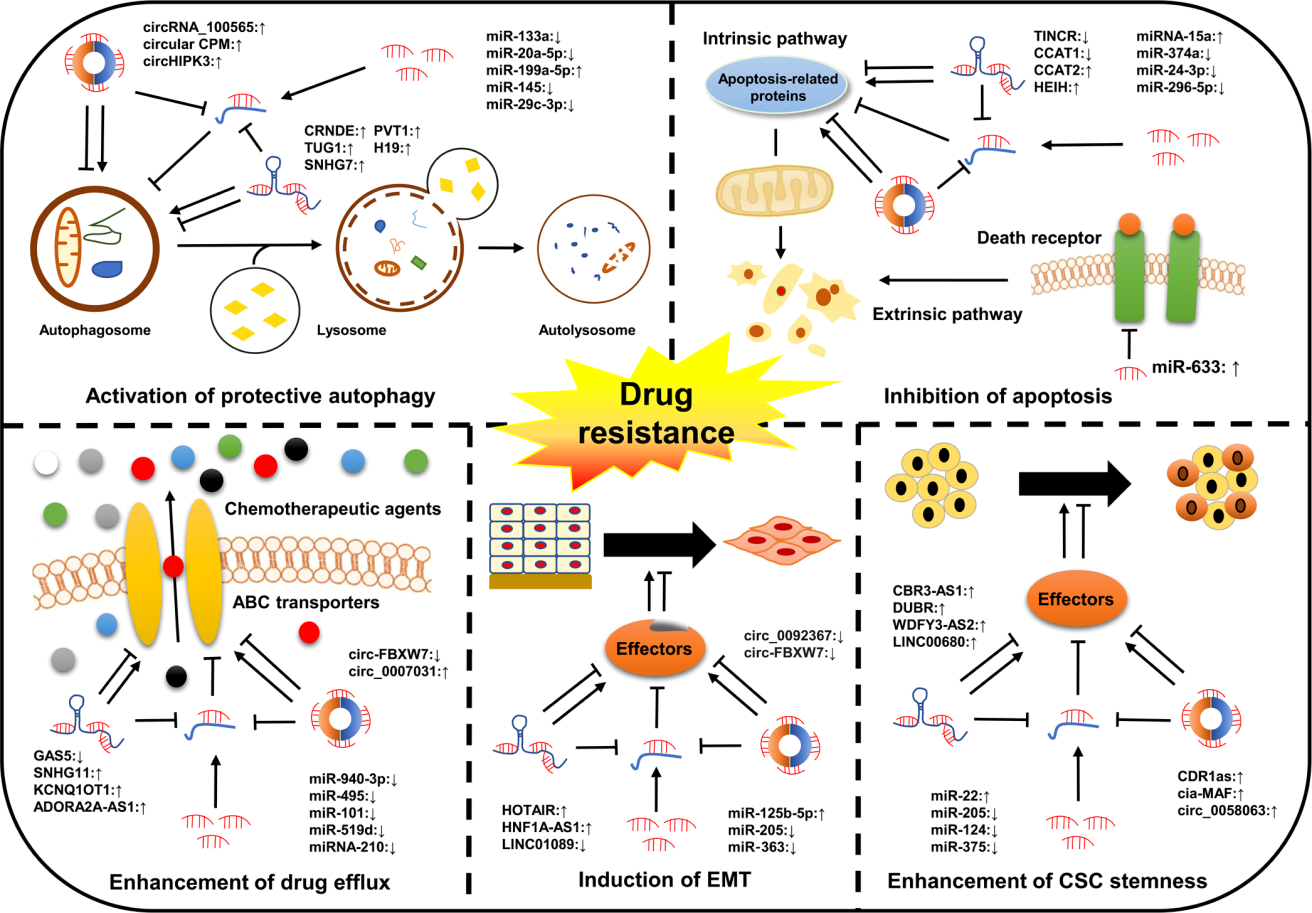
Classical mechanisms of ncRNAs in cancer drug resistance. The dysregulation of ncRNAs contributes to the development of cancer drug resistance by modulating multiple cellular processes of cancer cells, such as drug efflux, cell apoptosis, autophagy, and EMT as well as the acquisition of CSC characteristics.

Chemotherapy remains the most effective first-line therapeutic approach for all stages of cancer and can effectively improve the clinical outcomes of patients in the short term. However, its long-term role in extending the OS of cancer patients is extremely restricted due to the emergence of drug resistance (43). Drug resistance is classified into single drug resistance and multidrug resistance (MDR). Of these, MDR is the main cause of mortality for most patients (44). Emerging evidence has shown that ncRNAs are closely associated with cancer drug resistance (**Table 1**). Their dysregulation contributes to the development of cancer drug resistance *via* distinct mechanisms, including inhibition of apoptosis, activation of protective autophagy, enhancement of drug efflux, induction of EMT, and enhancement of cancer stem cells (CSCs) stemness (**Figure 2**). However, the exact mechanisms are still not fully clarified. In this section, the involvement of ncRNAs in cancer drug resistance is outlined.

**Table 1:** Roles of ncRNAs in cancers drug resistance.

Cancer types	Chemotherapeutic drugs	ncRNAs	Gene type	Alteration	Effect on Drug Resistance	Reference
OC	CDDP	miR-133a, miR-29c-3p, miR-30a LINC01125, LINC01508 circRNA Cdr1as	Tumor suppressor	Downregulated	Sensitivity to CDDP	(45–50)
		miR-181d, miR-149-3p lncRNA WDFY3-AS2, lncRNA CCAT1 circ-LPAR3, circHIPK2	Oncogene	Upregulated	Resistance to CDDP	(51–56)
	PTX	miR-194-5p, hsa‐miR‐105 lncRNA SNHG5, lncRNA KB-1471A8.2 circEXOC6B	Tumor suppressor	Downregulated	Sensitivity to PTX	(57–61)
		lncRNA SDHAP1, lncRNA HULC circ_0061140, circ_CELSR1	Oncogene	Upregulated	Resistance to PTX	(62–65)
GC	5-FU	miR-204, miR195, exosomal miR-107	Tumor suppressor	Downregulated	Sensitivity to 5-FU	(66–68)
		miR-149 lncRNA HAGLR, lncRNA HNF1A-AS1 circRNA CPM, circNRIP1	Oncogene	Upregulated	Resistance to 5-FU	(69–73)
	CDDP	microRNA-206 lncRNA ADAMTS9-AS2 circRNA MCTP2, circ_0001017	Tumor suppressor	Downregulated	Sensitivity to CDDP	(74–77)
		miR-193a-3p lncRNA BANCR, lncRNA MCM3AP-AS1 circRNA DONSON	Oncogene	Upregulated	Resistance to CDDP	(78–81)
	OXA	hsa_circ_0001546	Tumor suppressor	Downregulated	Sensitivity to OXA	(82)
		lncRNA DDX11-AS1 circ_0032821	Oncogene	Upregulated	Resistance to OXA	(83, 84)
NSCLC	CDDP	miR-186-5p, miR-101-3p lncRNA SPRY4-IT1 circ_0030998	Tumor suppressor	Downregulated	Sensitivity to CDDP	(85–88)
		microRNA-25-3p lncRNA SNHG1, LINC01224 circRNA_100565	Oncogene	Upregulated	Resistance to CDDP	(89–92)
CRC	5-FU	miR-375-3p lncRNA HAND2-AS1, circDDX17	Tumor suppressor	Downregulated	Sensitivity to 5-FU	(93–95)
		miR-29b-3p lncRNA LBX2-AS1 circ_0007031	Oncogene	Upregulated	Resistance to 5-FU	(96–98)
	OXA	miR-200b-3p circ-FBXW7	Tumor suppressor	Downregulated	Sensitivity to OXA	(99, 100)
		miR-454-3p lncRNA CACS15	Oncogene	Upregulated	Resistance to OXA	(101, 102)
HCC	sorafenib	miR-138-1-3p, miRNA-124-3p.1 lncRNA FOXD2‐AS1	Tumor suppressor	Downregulated	Sensitivity to sorafenib	(103–105)
		miR-126-3p lncRNA DANCR circFOXM1	Oncogene	Upregulated	Resistance to sorafenib	(106–108)
	CDDP	miR-27a-3p lncRNA GAS5	Tumor suppressor	Downregulated	Sensitivity to CDDP	(109, 110)
		lncRNA FGD5-AS1 circMRPS35	Oncogene	Upregulated	Resistance to CDDP	(111, 112)
BC	ADR	miR-3609 circKDM4C	Tumor suppressor	Downregulated	Sensitivity to ADR	(113, 114)
		microRNA-221 lnc-LOC645166 circRNA_0044556	Oncogene	Upregulated	Resistance to ADR	(115–117)
	tamoxifen	lncRNA ADAMTS9-AS2 hsa_circ_0025202	Tumor suppressor	Downregulated	Sensitivity to tamoxifen	(118, 119)
		miR-24-3p lncRNA CYTOR	oncogene	Upregulated	Resistance to tamoxifen	(120, 121)
CC	CDDP	miR-144	Tumor suppressor	Downregulated	Sensitivity to CDDP	(122)
		lncRNA OTUD6B-AS1	Oncogene	Upregulated	Resistance to CDDP	(123)
Prostate cancer	docetaxel	circFoxo3	Tumor suppressor	Downregulated	Sensitivity to docetaxel	(124)
		exosomal circ-XIAP	Oncogene	Upregulated	Resistance to docetaxel	(125)
PC	gemcitabine	miRNA-3662 circ_0092367	Tumor suppressor	Downregulated	Sensitivity to gemcitabine	(126, 127)
		miR-93-5p lncRNA PVT1 circHIPK3	Oncogene	Upregulated	Resistance to gemcitabine	(128–130)
Bladder cancer	CDDP	exosomal LINC00355 circ_0058063	Oncogene	Upregulated	Resistance to CDDP	(131, 132)
Renal cancer	sunitinib	miR-130b circSNX6	Oncogene	Upregulated	Resistance to sunitinib	(133, 134)

### MiRNAs and cancer drug resistance

#### MiRNAs affect drug-induced apoptosis by targeting apoptosis-related proteins or drug-resistance pathways

The inhibition of drug-induced apoptosis is one of the main mechanisms contributing to cancer drug resistance. Apoptosis can be divided into two categories: the extrinsic pathways mediated by death receptors and intrinsic (mitochondrial) pathways associated with apoptosis-related proteins such as B-cell lymphoma-2 (Bcl-2) (135). MiRNAs have been shown to influence cancer drug resistance by manipulating apoptosis-related proteins (27, 136). For instance, in our previous work, miR-633 was found to be significantly upregulated in GC tissues and cells, and its upregulation in GC samples was closely associated with the downregulation of Fas-associated protein with death domain (FADD), an adaptor involved in the extrinsic pathway of apoptosis. Mechanistic analysis revealed that miR-633 inhibited doxorubicin (DOX)/cisplatin (CDDP)-induced apoptosis in SGC-7901 and AGS cells by downregulating FADD *via* directly targeting its 3′-UTR (137). In another study, Yang et al. found that miR‐92a-3p was upregulated in both cervical cancer (CC) tissues and CDDP-resistant CC cell lines HeLa and SiHa. The overexpression of miR‐92a-3p inhibited the CDDP-induced apoptosis of HeLa and SiHa cells by targeting the expression of Krüppel-like factor 4 (KLF4), leading to the enhancement of CDDP resistance in CC. Consistent with this, miR‐92a-3p knockdown increased the sensitivity of HeLa and SiHa cells to CDDP (138).

The Bcl-2 family consisting of anti-apoptotic proteins (e.g., Bcl-2, Mcl-1, and Bcl-xl) and pro-apoptotic proteins (e.g., Bax, Bim, and Bak) plays crucial roles in the mitochondrial apoptotic pathway (139). It has been reported that the ratio between anti-apoptotic proteins and BH3-only proteins (a subtype of pro-apoptotic proteins) can alter the outer mitochondrial membrane permeability by regulating the activation of pore-forming proteins, thereby inducing apoptosis of the cancer cells (140). Zhong et al. showed that miR-625-3p overexpression significantly inhibited the CDDP-induced apoptosis of high-grade serous ovarian cancer (OC) cells OVCAR3 and OVCAR4 by targeting Bcl-2 and Bax expression, resulting in the inhibition of CDDP sensitivity in these cells (141). Ashofteh et al. revealed that miRNA-15a promoted cellular apoptosis by downregulating the mRNA levels of Mcl-1 and Bcl-2 in chronic lymphocytic leukemia (CLL), thereby enhancing the sensitivity of CLL-CII leukemia cells to fludarabine (142). In addition, Sun et al. demonstrated that miR‐374a was downregulated in A2780 cells by propofol. The overexpression miR‐374a suppressed the apoptosis of A2780 cells by decreasing the expression of Bim, p27, and FOXO1, leading to the enhancement of CDDP resistance in OC (143).

The Wnt/β-catenin signaling pathway is involved in the modulation of various cellular processes of cancer cells, such as apoptosis, proliferation, and metastasis. The dysregulation of this pathway has been shown to contribute to the development of cancer drug resistance by influencing the apoptotic pathways (144). For instance, Han et al. found that miR-199b-3p knockdown enhanced the sensitivity of cetuximab-resistant OC cells SW480 and HCT116 to cetuximab by promoting cell apoptosis. Mechanistically, silencing miR-199b-3p could enhance cetuximab-induced apoptosis in cetuximab-resistant SW480 and HCT116 cells by activating the Wnt/β-catenin signaling pathway *via* downregulating CRIM1 (145). Liu et al. showed that miR-217 was significantly reduced in CDDP-resistant OC COC1 cells compared with in CDDP-sensitive COC1 cells. The overexpression of miR-217 in COC1 cells facilitated CDDP-induced apoptosis and enhanced CDDP sensitivity by inhibiting the activation of the Wnt/β-catenin signaling pathway (146). In addition, multiple miRNAs, such as miR-323a-3p, miR-6727-5p, and miRNA-223-3p, have also been found to contribute to the development of cancer drug resistance by regulating apoptotic pathways *via* targeting other drug resistance-related signaling pathways, including the phosphatidylinositol 3-kinase (PI3K)/AKT, mitogen-activated protein kinase (MAPK), and nuclear factor kappa B (NF-κB) signaling pathways (147–149). Collectively, these findings indicate that targeting the apoptotic pathways is a common regulation mechanism for miRNAs in cancer drug resistance. Further investigating the mechanisms of miRNAs in drug-induced apoptosis may provide new insights on therapeutic strategies against cancer drug resistance.

#### MiRNAs and drug efflux in cancer drug resistance

Excessive drug efflux is considered a critical mechanism contributing to cancer drug resistance, in which the efficiency of anticancer drugs is significantly limited due to the reduction in drug concentration in cancer cells (150). It has been reported that excessive drug efflux is a result of the upregulation of drug efflux pumps, including ATP-binding cassette (ABC) transporters. Several members of the ABC family, such as ABCB1, ABCC2, and ABCG2, have been shown to contribute to the development of MDR in a variety of cancers (151). Increasing evidence suggest that miRNAs participate in the modulation of drug efflux in cancer cells by altering the expression ABC transporters (152). For instance, Zou et al. showed that the overexpression of miR-495 significantly reduced the drug efflux in MDR OC cell line A2780DX5 and GC cell line SGC7901R by directly targeting ABCB1, thereby enhancing the sensitivity of cancer cells to DOX and paclitaxel (PTX) (153). ABCB1 is also a target of miR-101 in GC. The overexpression of miR-101 in drug-resistant SGC7901 cells significantly decreased ABCB1 expression at the mRNA and protein levels (154). Tian et al. found that miR-940-3p negatively modulated ABCC2 expression in CDDP-resistant OVCAR3 and SKOV3 cells by directly binding to its 3’-UTR region, leading to the enhancement of CDDP sensitivity in OC (155). Additionally, Tsai et al. revealed that miR-519d was downregulated in human osteosarcoma cells MG-63 and U-2 by CCN family member 2, and its reduction facilitated drug resistance by upregulating the ABCG2 levels (156). Moreover, Amponsah et al. demonstrated that miR-210 overexpression decreased ABCC5 mRNA levels in pancreatic cancer (PC) cell lines (ASAN-PaCa, AsPC-1 and MIA-PaCa2) by targeting its 3’-UTR, leading to the enhancement of gemcitabine sensitivity in PC (157). In addition, several miRNAs, such as miR-34a, miR-7-5p, and miR-325-3p, have also been shown to play a role in cancer drug resistance by influencing drug efflux *via* targeting ABC transporters in a variety of cancers, including colon cancer, glioblastoma, and hepatocellular carcinoma (HCC) (158–160). Taken together, these studies strongly suggest that miRNAs are involved in the development of cancer drug resistance by altering the function of drug efflux pumps. However, the detailed mechanisms are still inconclusive and need to be further elucidated.

#### MiRNAs are involved in the development of cancer drug resistance by modulating autophagy

Autophagy is a lysosomal degradation process that is essential for cellular survival, differentiation, and homeostasis. Protective autophagy has been recognized as one of the main mechanisms resulting in cancer drug resistance, by which cancer cells eliminate the cytotoxicity of chemotherapeutic drugs (161). Therefore, targeting autophagy may be an effective therapeutic strategy for improving the poor prognosis of cancer patients. MiRNAs have been shown to participate in cancer drug resistance by regulating autophagy-related genes (162). For instance, Zhou et al. found that miR-133a was significantly downregulated in CDDP-resistant OC cell lines A2780 and SKOV3. The overexpression of miR-133a significantly enhanced the CDDP sensitivity in CDDP-resistant A2780 and SKOV3 cells by inhibiting autophagy *via* directly targeting YES1 (45). Li et al. showed that miR-20a-5p inhibited autophagy in CDDP-resistant OC cells. Mechanistically, miR-20a-5p suppressed the expression of RBP1 in CDDP-resistant A2780 and COC1 by promoting DNMT3B-mediated RBP1 methylation, resulting in the inhibition of autophagy and CDDP resistance in OC (163). H446/EP was a MDR small cell lung cancer (SCLC) cell line that was developed from H446. Li et al. demonstrated that miR-199a-5p was significantly upregulated in H446/EP cells compared to non-drug-resistant H446 cells. MiR-199a-5p overexpression decreased the CDDP sensitivity in H446 cells by enhancing the autophagy activity *via* directly targeting p62 (164). Moreover, Zhao et al. revealed that miR-145 was downregulated in CRC tissues and cell lines (HCT116, SW620, and HCT-8). The overexpression of miR-145 enhanced 5-fluorouracil (5-FU) sensitivity in 5-FU-resistant HCT116, SW620, and HCT-8 cells by enhancing 5-FU-induced apoptosis and reducing autophagy. Mechanistically, miR-145 activated p53 by directly targeting HDAC4, thereby inhibiting 5-FU resistance in CRC (165). Collectively, these studies indicate that protective autophagy induced by miRNA dysregulation is a crucial factor resulting in the occurrence of cancer drug resistance. Moreover, miRNAs may simultaneously target apoptotic pathways and autophagy. Thus, it is a valuable strategy to comprehensively identify miRNAs associated with these death pathways to help patients overcome cancer drug resistance.

#### MiRNAs alter stemness characteristics and EMT in cancer cells

CSCs, also known as tumor-initiating cells (TICs), are a unique subset of tumor cells exhibiting capabilities of self-renewal, differentiation, and tumor initiation. CSCs have been recognized as the main cause of drug resistance, metastasis, and recurrence of cancer (166, 167). Growing evidence suggests that miRNAs are involved in cancer drug resistance by altering the characteristics of CSCs (168). For instance, Zhang et al. found that miR-132 was upregulated in the Lrg5^+^ gastric CSCs isolated from MKN45 and MKN28 cells. High miR-132 expression was closely associated with chemo-resistance in GC patients. Mechanistic assays revealed that miR-132 facilitated CDDP resistance in Lrg5^+^ gastric CSCs by upregulating ABCG2 *via* directly targeting SIRT1 (169). Feng et al. showed that miR-25 was upregulated in liver CSCs (LCSCs) isolated from HepG2, Huh7, and PLC cells compared with the non-CSCs. The knockdown of miR-25 significantly enhanced the sensitivity of the LCSCs to tumor necrosis factor-related apoptosis-inducing ligand (TRAIL)-induced apoptosis by inhibiting Bad phosphorylation *via* upregulating phosphatase and tensin homologue (PTEN), a PI3K inhibitor (170). In addition, Ni et al. revealed that miR-375 suppressed the stemness of GC cells BGC-823 and SGC-7901 by triggering ferroptosis *via* directly targeting SLC7A11 (171). Epithelial mesenchymal transition (EMT) is a morphogenetic process that endows epithelial cells with migratory and invasive characteristics. The aberrant activation of EMT has been shown to facilitate the development of cancer drug resistance by enabling the conversion of non-CSCs into CSCs (172, 173). MiRNAs can participate in the development of cancer drug resistance by targeting the EMT process. For instance, Hirao et al. showed that the overexpression of miR-125b-5p in HCC cell lines (PLC/PRF5-R1/R2) enhanced sorafenib resistance. Mechanistically, miR-125b-5p promoted the EMT process and conferred stemness characteristics in PLC/PRF5-R1/R2 cells by targeting ATXN1, leading to the reduction of sorafenib sensitivity in HCC. Consistent with this, ATXN1 knockdown in HCC cells exhibited a higher CSC population and an EMT phenotype (174). Chaudhary et al. revealed that miR-205 was highly downregulated in gemcitabine-resistant MIA PaCa-2R cells compared to gemcitabine-sensitive MIA PaCa-2 cells. The overexpression of miR-205 resulted in a reduction in EMT, CSCs, and chemo-resistance markers in MIA PaCa-2R cells, suggesting that miR-205 can enhance the sensitivity of gemcitabine-resistant PC cells to gemcitabine (175). In addition, the overexpression of miR‐363 in drug-resistant OC cell lines (A2780cp and C13) restores CDDP sensitivity by directly targeting Snail (a mesenchymal marker). Consistent with this, Snail overexpression dramatically suppressed the effect of miR‐363 on CDDP resistance of A2780cp and C13 cells, indicating that miR‐363 regulates CDDP resistance in OC through Snail‐induced EMT (176). In summary, understanding the effect of miRNAs on stemness properties and EMT in the development of cancer drug resistance may provide new insights into the development of therapeutic strategies for patients with a poor response to chemotherapeutic agents.

#### MiRNAs are involved in the regulation of inflammation by targeting T cells

Chronic inflammation triggered by infections, aberrant immune reactions or environmental factors is an uncontrolled inflammatory response and contributes to cancer progression by influencing various biological behaviors of cancer cells, including cellular proliferation, invasion, angiogenesis, metastasis, and drug resistance (177). T cells are the major effector cells in cellular immunity. They are involved in inflammation by producing cytokines in immune responses (178). The immune evasion and immune tolerance induced by the dysregulation of T cell function have shown to be the main causes of drug resistance development (27). Therefore, targeting T cells is an effective way to improve drug sensitivity for cancer patients. An increasing amount of evidence suggests that miRNAs are crucial regulators of T cell functions. For example, Yan et al. discovered that miR-181a was upregulated in T-cell lymphoblastic lymphoma Jurkat and H9 cells treated with DOX, CDDP, cyclophosphamide, and cytarabine. Knockdown of miR-181a in Jurkat and H9 cells significantly enhanced the sensitivity of these chemotherapeutic drugs (179). Ning et al. demonstrated that miR-208b was upregulated in exosomes from CRC cell lines NCM460, SW480, and oxaliplatin (OXA)-resistant SW480. Exosomal miR-208b facilitated regulatory T cells expansion by targeting programmed cell death factor 4, thereby enhancing OXA resistance in CRC (180). Xu et al. showed that miR-424 (322) reversed drug resistance in OC by activating T cell immune response, resulting in the inhibition of immune evasion in drug-resistant OC. Mechanistically, miR-424 (322) inhibited IFN-γ-induced apoptosis in PD-L1-associated CD8^+^ T cells and altered T cell cytokine secretions by downregulating PD-L1, resulting in the enhancement of chemotherapy efficacy in Skov3 (CP) cells (181). In addition, the downregulation of miR-145 by CDDP in A2780 cells increased PD-L1 levels by directly targeting c-Myc, leading to the induction of T cell apoptosis and enhancement of CDDP resistance in OC (182), indicating that miR-145 dysregulation contributes to the development CDDP resistance *via* T cell dysfunction-mediated immune tolerance. All these findings support the hypothesis that miRNAs are involved in the regulation of cancer drug resistance by targeting T cells. Therefore, in-depth investigations are required to clarify the detailed mechanisms of miRNAs in regulating T cells, which may provide new insights into the development of miRNA-based therapeutic strategies for cancer patients, particularly those with a poor response to chemotherapy.

### LncRNAs and cancer drug resistance

#### LncRNAs control the cellular death pathways in cancer drug resistance

The dysregulation of lncRNAs has been shown to participate in the development of cancer drug resistance through interference with cellular apoptosis or proliferation pathways (18, 183). For instance, Li et al. revealed that lncRNA TINCR was significantly increased in CDDP-resistant choroidal melanoma (CM) tissues and cells. TINCR overexpression in OCM-1 cells promoted proliferation and inhibited apoptosis by upregulating ERK-2 *via* sponging miR-19b-3p, leading to the enhancement of CDDP resistance in CM (184). Zhou et al. found that lncRNA CCAT2 was upregulated in breast cancer (BC) tissues and 5-FU-resistant BC cell lines (MDA‐MB‐231, SKBR‐3, MCF‐7, and HCC‐1937) after chemotherapy. CCAT2 overexpression in 5-FU-resistant MDA‐MB‐231, MCF‐7 cells inhibited apoptosis and increased proliferation by activating the mTOR signaling pathway, resulting in a reduction in 5-FU sensitivity (185). Guo et al. demonstrated that lncRNA HEIH was upregulated in PTX-resistant endometrial cancer Ishikawa and HHUA cells. The overexpression of HEIH in Ishikawa and HHUA cells enhanced PTX resistance by depressing cell apoptosis and enhancing cell proliferation and viability *via* activating the MAPK signaling pathway (186). Zhu et al. showed that LINC00942 was significantly upregulated in drug-resistant GC cell lines SGC7901 and BGC823, and its overexpression in SGC7901 and BGC823 cells facilitated drug resistance by suppressing cellular apoptosis and enhancing their stemness features. Mechanistically, LINC00942 upregulated MSI2 by inhibiting its degradation *via* preventing its interaction with SCFβ-TRCP E3 ubiquitin ligase, thereby stabilizing c-Myc mRNA in an m6A-dependent manner (187). In addition, several oncogenic lncRNAs, such as APOC1P1-3, PRLB, and WDFY3-AS2, have also been reported to promote drug resistance by targeting the apoptotic pathways in distinct cancer types (53, 188, 189).

Recent studies indicate that the activation of autophagy by chemotherapeutic agents can protect cancer cells from drug-induced apoptosis (161, 190). Zhang et al. showed that exosomal lncRNA SNHG7 was highly expressed in docetaxel-resistant lung adenocarcinoma (LUAD) H1299 and SPC-A1 cells. The knockdown of SNHG7 in docetaxel-resistant H1299 and SPC-A1 cells significantly inhibited cell proliferation and autophagy and enhanced docetaxel sensitivity. Mechanistically, SNHG7 upregulation facilitated autophagy of H1299 and SPC-A1 cells by stabilizing autophagy-related genes autophagy related 5 (ATG5) and autophagy related 12 (ATG12) *via* recruiting human antigen R (HuR), resulting in the enhancement of docetaxel resistance in LUAD. Moreover, the transmission of exosomal SNHG7 from docetaxel-resistant H1299 and SPC-A1 cells to parental H1299 and SPC-A1 cells also promoted docetaxel resistance (191). In another study, lncRNA TUG1 was found to be upregulated in CRC tissues. The overexpression of TUG1 in LoVo and HCT15 cells enhanced CDDP resistance. Functional assays revealed that TUG1 promoted the proliferation and autophagy of LoVo and HCT15 cells by activating the HDGF/DDX/β-catenin axis *via* sequestrating miR-195-5p, leading to the enhancement of CDDP resistance in CRC (192). In addition, Chen et al. found that CRNDE triggered autophagy in HepG2 and Hep3B cells by increasing ATG4B levels *via* sponging miR-543. CRNDE silencing enhanced the sorafenib sensitivity of HepG2 and Hep3B cells, indicating that CRNDE may promote sorafenib resistance in HCC by driving ATG4B-mediated autophagy (193). High-mobility group box 1 (HMGB1) is a classical non-histone protein closely associated with autophagy (194). Chen et al. revealed that lncRNA H19 overexpression in CDDP-resistant TU-177 and AMC-HN-8 cells significantly facilitated autophagy by upregulating HMGB1 *via* sequestrating miR-107, resulting in the enhancement of CDDP resistance in laryngeal squamous cell carcinoma (LSCC). Consistent with this, the knockdown of H19 in CDDP-resistant TU-177 cells inhibited autophagy and CDDP resistance (195). Taken together, these findings strongly suggest that lncRNAs are widely involved in the development of cancer drug resistance by targeting cellular death pathways. However, the detailed mechanisms are still not fully understood; additional investigations are required to fully uncover the regulatory role of lncRNAs in cellular death pathways.

#### LncRNAs modulate ABC transporter-mediated drug efflux in cancer cells

The upregulation of ABC transporters is considered a main cause of MDR development in cancer. An increasing amount of evidence has shown that lncRNAs are involved in cancer drug resistance by regulating ABC transporter-mediated drug efflux (196). For instance, Chen et al. revealed that lncRNA GAS5 overexpression in BC cells significantly enhanced the adriamycin (ADR) sensitivity by inhibiting ABCB1-mediated drug efflux. Mechanistically, GAS5 suppressed the expression of ABCB1 in ADR-resistant MCF-7 cells by activating the Wnt/β-catenin signaling pathway *via* miR-221-3p/DKK2 axis (197). In another study, lncRNA ADORA2A-AS1 was found to be upregulated in chronic myeloid leukemia (CML). ADORA2A-AS1 knockdown in K562 and KCL22 cells significantly enhanced the imatinib sensitivity of cells. Functional assays showed that ADORA2A-AS1 facilitated ABCC2 expression in K562 and KCL22 cells *via* sponging miR-665, indicating that ADORA2A-AS1 may contribute to the development of imatinib resistance by driving ABCC2-mediated drug efflux in CML (198). Moreover, Wang et al. demonstrated that lncRNA KCNQ1OT1 significantly increased in temozolomide (TMZ)-resistant U251 and U87 cells compared to TMZ-sensitive U251 and U87 cells. KCNQ1OT1 overexpression in TMZ-resistant U251/TMZ and U87/TMZ cells significantly upregulated the expression of ABCB1, c-Myc, and survivin by increasing PIM1 expression *via* sponging miR-761, leading to the enhancement of TMZ resistance (199). Shen et al. found that lncARSR was upregulated in ADR-resistant osteosarcoma U2OS and MG63 cells and accompanied by acquired MDR against PTX and CDDP. Mechanistically, lncARSR overexpression in ADR-resistant U2OS and MG63 cells significantly promoted cell rhodamine 123 efflux, survival, and migration by upregulating ABCB1, survivin, and matrix metalloproteinase-2 (MMP2) *via* activating AKT. Consistent with this, lncARSR knockdown in these ADR-resistant osteosarcoma cells facilitated cell rhodamine 123 retention and apoptosis (200). In addition, Li et al. showed that lncRNA HOTTIP was highly expressed in serum from esophageal cancer (EC) patients. Extracellular vesicles-containing HOTTIP contributed to ADR resistance in EC Eca109 cells by positively activating ABCG2 (201). Collectively, these studies indicate that the dysregulation of lncRNAs contributes to the development of cancer drug resistance by modulating ABC transporter-mediated drug efflux *via* targeting miRNAs. The exact mechanisms of the lncRNA/miRNA axis in drug efflux need to be further elucidated.

#### LncRNAs manipulate malignant features of cancer cells

LncRNAs have been shown to regulate the stemness of cancer cells, thereby demonstrating their regulatory roles in cancer drug resistance. For instance, Xie et al. found that lncRNA CBR3-AS1 was significantly upregulated in CRC cell lines (HCT116, HT29, SW620, and SW480) compared to normal colon epithelial FHC cells. CBR3-AS1 knockdown in OXA-resistant HCT116 and SW480 cells notably enhanced OXA sensitivity. Mechanistically, CBR3-AS1 knockdown inhibited the stem-like properties of HCT116 and SW480 cells by downregulating Nanog, Sox2, and Oct4 (stem cell markers) *via* sponging miR-145-5p, resulting in the reduction of OXA resistance in CRC (202). Liu et al. showed that lncROPM promoted the drug resistance of breast CSCs (BCSCs) isolated from BT-549, Hs578T, and MCF-7 cells by upregulating PLA2G16 *via* increasing its mRNA stability. Moreover, lncROPM contributed to the maintenance of BCSC stemness by facilitating phospholipid metabolism and the production of free fatty acid (such as arachidonic acid) *via* increasing the PLA2G16 levels (203). Cheng et al. revealed that lncRNA SNHG7 significantly increased in PC cells (PANC-1 and AsPC-1) co-cultured with mesenchymal stem cells (MSCs). The upregulation of SNHG7 induced by the MSCs in PANC-1 and AsPC-1 cells facilitated stemness of cells and Folfirinox resistance by activating the Notch1/Jagged1/Hes-1 signaling pathway *via* increasing Notch1 expression (204). In addition, Liu et al. demonstrated that lncRNA DUBR was highly expressed in HCC tissues and liver CSCs isolated from MHCC-97H, SNU-368 and MIHA, and its high expression was closely associated with poor chemotherapy response. DUBR overexpression in SNU-368 and MHCC-97H cells promoted the stemness of cancer cells and OXA resistance. Functional assays revealed that DUBR activated the Notch1 signaling pathway by upregulating cancerous inhibitor of protein phosphatase 2A (CIP2A) levels *via* sponging miR-520d-5p, leading to the enhancement of the stemness characteristics of the HCC cells and drug resistance (205).

LncRNA dysregulation contributes to the development of cancer drug resistance by altering T cell activity. For instance, KCNQ1OT1 was found to be upregulated in sorafenib‐resistant HCC tissues and cells, and its knockdown in sorafenib‐resistant SK-HEP-1 and Huh-7 cells co-cultured with T cells significantly inhibited immune escape by enhancing the immune surveillance ability of T cells. Mechanistically, KCNQ1OT1 upregulated PD‐L1 levels in sorafenib‐resistant SK-HEP-1 and Huh-7 cells by sponging miR‐506, thereby reducing the apoptosis of CD8^+^ T cells (206). In another study, LINC00184 overexpression in docetaxel-resistant DU145 and PC3 cells facilitated cell immune escape by upregulating PD-L1 *via* sponging miR-105-5p, resulting in the enhancement of docetaxel resistance in PCa (207). In addition, HCG18 could inhibit CD8^+^ T cells activity by increasing PD-L1 levels *via* sponging miR-20b-5p, leading to the promotion of cetuximab resistance in CRC cells (208). LncRNAs can also act as the recruiters of epigenetic modifiers to play a role in cancer drug resistance. For instance, Li et al. found that PCAT-1 was upregulated in CDDP-resistant GC tissues and cell lines. PCAT-1 knockdown resensitized CDDP-resistant BGC823 and SGC790 cells to CDDP. Functional assays revealed that PCAT-1 epigenetically silenced PTEN by increasing H3K27me3 *via* recruiting the histone methyltransferase enhancer of zeste homolog 2 (EZH2), resulting in the enhancement of CDDP resistance in GC (209). Si et al. showed that H19 was highly expressed in PTX-resistant BC cells. H19 upregulation in PTX-resistant MCF-7 and ZR-75-1 cells facilitated the recruitment of EZH2 to the *BIK* gene promoter, increasing H3K27me3 modification and suppressing *BIK* gene expression (210). In addition, Lin et al. revealed that LINC00261 was downregulated in 5-FU-resistant EC tissues. The overexpression of LINC00261 dramatically inhibited resistance to apoptosis in 5-FU-resistant TE-1 and -5 cells, whereas LINC00261 knockdown observed the opposite effect. Mechanistically, LINC00261 significantly decreased the levels of dihydropyrimidine dehydrogenase by increasing the methylation of its promoter through the recruitment of DNA methyltransferase, thereby enhancing 5-FU sensitivity in EC (211).

LncRNAs are able to govern the EMT process and malignant features of cancer cells to play a role in cancer drug resistance. Li et al. discovered that HOTTIP overexpression in glioma A172 and LN229 cells significantly increased cell proliferation, migration, and metastasis. Further, HOTTIP facilitated the EMT process in TMZ-resistant A172 and LN229 cells by decreasing E-cadherin expression and increasing Zeb1/Zeb2 (mesenchymal markers) *via* upregulating miR-10b, resulting in the enhancement of TMZ resistance in glioma. Consistent with this, miR-10b knockdown in HOTTIP-overexpressing A172 and LN229 cells reversed the EMT with associated TMZ sensitization (212). Zhang et al. demonstrated that HOTAIR facilitated migration, proliferation, and the resistance of HeLa and Siha cells to CDDP, PTX, and docetaxel. Mechanistically, HOTAIR enhanced the EMT process in HeLa and Siha cells by activating the PTEN/PI3K axis *via* sequestrating miR-29b, leading to the enhancement of MDR in CC (213). Jiang et al. revealed that HNF1A-AS1 facilitated 5-FU resistance in GC cells (MKN-45 and HGC-27) by enhancing the EMT process *via* increasing EIF5A2 levels. HNF1A-AS1 served as a sponge of miR-30b-5p to upregulate EIF5A2 (71). Moreover, Zhao et al. showed that DLX6-AS1 promoted proliferation, migration, invasion, and secondary CDDP resistance in LSCC cell lines SK-MES-1 and NCIH226. Mechanistically, DLX6-AS1 increased the expression of CUGBP, Elav-like family member 1 by sponging miR-181a-5p and miR-382-5p, resulting in the secondary CDDP resistance of LSCC cells (214). In addition, multiple lncRNAs, such as LINC01089, CYTOR, and H19, have also been shown to demonstrate their roles in cancer drug resistance by targeting the EMT process and altering malignant characteristics, such as proliferation, invasion, and metastasis (215–217). Altogether, these findings suggest that the underlying mechanisms of lncRNAs in cancer drug resistance involve their modulation of CSC expansion, T cell activity, EMT process, and malignant characteristics. In-depth investigations are required to fully elucidate the exact mechanisms behind lncRNA-mediated cancer drug resistance, which will be of great benefit in the development of lncRNA-based therapeutic strategies for cancer patients exhibiting a poor response to chemotherapy.

### CircRNAs and cancer drug resistance

In recent years, the role of circRNAs in cancer progression has become a research hotspot, but the investigation of the contribution of circRNAs to cancer drug resistance is still at an initial stage (20, 218, 219). Emerging evidence indicates that the dysregulation of circRNAs is involved in cancer drug resistance *via* distinct mechanisms, such as drug transportation, cell death, DNA repair, and cancer stemness (220).

CircRNAs mainly act as miRNA sponges to play regulatory roles in cancer drug resistance. For instance, Xu et al. showed that circ-FBXW7 was downregulated in OXA-resistant CRC tissues and cells. Exosomal transfer of circ-FBXW7 enhanced the sensitivity of the OXA-resistant SW480 and HCT116 cells to OXA by inhibiting OXA efflux, elevating the OXA-induced apoptosis, and suppressing OXA-induced EMT *via* sponging miR-18b-5p (100). Another circRNA, circRNA_101277, was found to be highly expressed in CRC tissues and cells, and its overexpression in SW620 and SW480 cells facilitated CDDP resistance by upregulating IL-6 *via* sequestering miR-370 (221). Furthermore, Zhong et al. revealed that circRNA_100565 was upregulated in CDDP-resistant NSCLC tissues and cells. CircRNA_100565 knockdown in the drug-resistant A549 and H1299 cells reduced CDDP resistance by enhancing cell apoptosis and inhibiting proliferation and autophagy. Mechanistically, circRNA_100565 exerted its anti-drug resistant role by upregulating ADAM28 expression *via* sponging miR-377-3p in CDDP-resistant A549 and H1299 cells (92). Additionally, Huang et al. demonstrated that circAKT3 was highly expressed in CDDP-resistant GC tissues and cells compared to CDDP-sensitive samples. The upregulation of circAKT3 was closely associated with aggressive characteristics in GC patients receiving CDDP treatment. Functional assays demonstrated that circAKT3 upregulated PIK3R1 *via* sequestrating miR-198, thereby enhancing CDDP resistance by facilitating DNA damage repair and inhibiting the apoptosis of CDDP-resistant SGC7901 and BGC823 cells (222). CircRNA CDR1as was found to contribute to the development of CDDP resistance in NSCLC by altering the stemness characteristics of NSCLC cells. The overexpression of circRNA CDR1as in CDDP-sensitive NSCLC cells (A549, H1299, and Calu6) significantly increased the expression of stemness signatures (e.g., Sox2, Oct4 and Nanog) by upregulating HOXA9 *via* sponging miR-641, leading to the enhancement of CDDP resistance. Consistent with this, circRNA CDR1as knockdown in CDDP-resistant A549, H1299, and Calu6 cells suppressed the stemness of cancer cells (223). Moreover, Huang et al. demonstrated that circ_0001598 was highly expressed in trastuzumab-resistant BC samples, and its overexpression facilitated immune escape and trastuzumab-resistance of SKBR-3 and BT474 cells by upregulating PD-L1 levels *via* sponging PD-L1 (224). In addition, Chen et al. showed that high circUSP7 levels are closely associated with CD8^+^ T cell dysfunction in NSCLC patients. Exosomal circUSP7 inhibited CD8^+^ T cell activity by upregulating Src homology region 2 (SH2)-containing protein tyrosine phosphatase 2 *via* sponging miR-934, resulting in enhanced resistance to anti-PD1 immunotherapy in NSCLC patients (225). There is no doubt that circRNAs have multifaceted functions in cancer drug resistance due to the broad involvement of miRNAs.

CircRNAs can also participate in cancer drug resistance by combining with other molecules. For instance, Wei et al. found circ0008399 enhanced CDDP resistance in bladder cancer EJ and T24T cells by upregulating TNF alpha-induced protein 3 (TNFAIP3) *via* directly binding to Wilms’ tumor 1-associating protein (WTAP). Mechanistically, circ0008399 interacted with WTAP to promote the formation of the WTAP/METTL3/METTL14 m6A methyltransferase complex, thereby upregulating TNFAIP3 expression in an m6A-dependent manner. Consistent with this, targeting the circ0008399/WTAP/TNFAIP3 axis promoted CDDP sensitivity in EJ and T24T cells (226). Hu et al. showed that circFARP1 was involved in the regulation of stemness and gemcitabine resistance in pancreatic ductal adenocarcinoma by altering the ability of cancer-associated fibroblasts *via* leukemia inhibitory factor (LIF). Functional assays revealed that circFARP1 directly interacted with caveolin 1 to inhibit its degradation by blocking the binding of caveolin 1 to its ubiquitin E3 ligase zinc and ring finger 1 (ZNRF1), thereby enhancing LIF secretion (227). In addition, Chen et al. demonstrated that the overexpression of circRNA cia-MAF drove LCSC propagation, self-renewal, and metastasis by facilitating MAFF expression *via* recruiting the TIP60 complex to its promoter, indicating that cia-MAF may contribute to the drug resistance of liver cancer by modulating CSCs (228). Particular circRNAs may participate in cancer drug resistance by altering the key regulators during cancer progression. Further investigations are required to fully understand the detailed mechanisms of circRNAs in cancer drug resistance. In addition, the circRNA/miRNA axis associated with chemotherapeutic responsiveness in cancer should be clarified.

### PiRNAs and cancer drug resistance

PiRNAs are a novel class of short chain ncRNAs (26-30 nucleotides) involved in a wide variety of physiological and pathological processes. They can regulate the expression of somatic genes through various mechanisms, including DNA methylation, chromatin modification and transposon silencing (229, 230). An increasing amount of evidence suggests that piRNAs are key regulators in the development of cancer drug resistance (231–233). For instance, Tan et al. discovered that piRNA-36,712 was significantly downregulated in BC tissues. The overexpression of piRNA-36,712 in MCF-7 and ZR75-1 cells significantly enhanced the sensitivity of cells to PTX and DOX. Correspondingly, piRNA-36,712 knockdown obtained the opposite effects. Mechanistically, piRNA-36,712 directly interacted with *SEPW1P* RNA (SEPW1 pseudogene), thereby suppressing SEPW1 expression by facilitating miR-7 and miR-324 to target *SEPW1* RNA, resulting in the enhancement of PTX and DOX sensitivity in BC (231). Mai et al. showed that piRNA-54265 was upregulated in CRC tissues. The overexpression of piRNA-54265 in HCT116 and LoVo cells promoted the formation of PIWIL2/STAT3/p-SRC complex by directly binding to PIWIL2, thereby activating the STAT3 signaling pathway, leading to the resistance of CRC cells to 5-FU and OXA (232). In addition, Wang et al. demonstrated that piR-L-138 was upregulated in CDDP-treated LSCC cells and patient-derived xenograft treated with CDDP. The knockdown of piR-L-138 in H157 and SKMES-1 cells enhanced CDDP sensitivity by directly binding to p60-MDM2 (233). Collectively, these findings indicate that piRNAs play vital roles in the regulation of cancer drug resistance, but the detailed mechanisms remain largely unknown. In-depth investigation may bring great benefits to the development of piRNA-based therapeutic strategies for cancer patients, particularly those with a poor response to chemotherapy.

## Clinical implications of ncRNAs in cancer drug resistance

### NcRNAs as biomarkers for the diagnosis and prognosis of cancer patients

It has been reported that approximately 50% of cancer patients are diagnosed at an advanced stage, with poor response rates and a low chance of cure (3). This is the main factor leading to the poor survival of cancer patients. Moreover, it is difficult for most cancer patients to obtain accurate individualized therapeutic strategies due to the lack of effective methods for prognostic assessment in clinical practice. In recent years, several protein biomarkers, such as carcinoembryonic antigen, carbohydrate antigen 15-3, and human epidermal growth factor receptor-2, have been applied in the early diagnosis and prognostic assessment of cancer patients. However, the unsatisfactory sensitivity and specificity of these biomarkers restricts their further utilization (234–236). Thus, it is urgent to develop new biomarkers with high sensitivity and specificity for cancer patients, particularly those with a poor response to chemotherapy.

NcRNAs can be secreted in actively packed particles (e.g., exosomes, microvesicles, or apoptotic bodies) and freely circulate in the blood, and their concentrations are almost the same as those in primary tumors (237, 238). Moreover, they also exhibit some unique characteristics, such as differently expressed patterns, high stability, and high detectability (239). These features strongly suggest that ncRNAs possess great potential as ideal diagnostic and prognostic biomarkers for cancer patients in clinical treatment. In fact, a large number of ncRNAs, particularly miRNAs and lncRNAs, have been identified as diagnostic and/or prognostic biomarkers of cancer (**Table 2**). For instance, Pan et al. showed that the levels of miR-33a-5p and miR-128-3p in whole blood were significantly downregulated in lung cancer patients or early-stage lung cancer patients compared to healthy controls. Further prospective study revealed that the area under the curve (AUC) value for the combination of miR-33a-5p and miR-128-3p was 0.9511, which was higher than that for CYFR21-1 (0.5856), NSE (0.6189), and CA72-4 (0.5206), indicating that the combination of the two miRNAs can serve as novel biomarkers for the early detection of lung cancer (284). In another study, Lu et al. developed a 21-miRNA-based diagnostic model and a 3-miRNA-based prognostic model that can be used to predict the prognosis of uterus corpus endometrial cancer patients and their response to chemotherapy and immunotherapy. The AUC values for the diagnostic panels were 0.911 in the training set, 0.827 in the test set, and 0.878 in the entire set. The diagnostic panel was closely associated with tumor mutation burden, PDL1 expression, and the infiltration of immune cells. Moreover, the prognostic risk signature of the prognostic panel can be used to predict the response to some commonly used chemotherapy regimens (285).

**Table 2:** NcRNAs as biomarkers diagnostic and prognostic in cancers drug resistance.

Cancer types	Biomarker types	ncRNAs	Potential values	Reference
OC	Diagnosis	miR-138-5p, miR-182-5p LINC01508 circRNA_0000735	Low levels of miR-138-5p, miR-182-5p, LINC01508 and circRNA_0000735 predict poor response to chemotherapy.	(49, 240–242)
		miR-205-5p lncRNA CHRF exosomal circFoxp1	High levels of miR-205-5p, lncRNA CHRF and exosomal circFoxp1 predict poor response to chemotherapy.	(243–245)
	Prognosis	miR-378a-3p, miR-513a-3p LINC00515	Low levels of miR-378a-3p, miR-513a-3p and LINC00515 predict poor prognosis.	(246–248)
		miR-98-5p lncRNA HOTAIR circTNPO3	High levels of miR-98-5p, lncRNA HOTAIR and circTNPO3 predict poor prognosis.	(249–251)
GC	Diagnosis	miR-124-3p lncRNA CASC2 hsa_circ_0000520	Low levels of miR-124-3p, lncRNA CASC2, hsa_circ_0000520 predict poor response to chemotherapy.	(252–254)
		exosomal miR-223 lncRNA MALAT1 circ_0026359	High levels of exosomal miR-223, lncRNA MALAT1, circ_0026359 predict poor response to chemotherapy.	(255–257)
	Prognosis	miR-34a hsa_circ_0001546	Low levels of miR-34a and hsa_circ_0001546 predict poor prognosis.	(82, 258)
		miR-15a-5p lncRNA EIF3J-DT circ_0026359	High levels of miR-15a-5p, LncRNA EIF3J-DT and circ_0026359 predict poor prognosis.	(257, 259, 260)
NSCLC	Diagnosis	miR-519d-3p	Low expression level of miR-519d-3p correlates with a decreased responsiveness to gefitinib.	(261)
		exosomal miR-136-5p lncRNA HOST2	High level of exosomal miR-136-5p and lncRNA HOST2 predict poor response to chemotherapy.	(262, 263)
	Prognosis	miR‐133a‐3p lncRNA RHPN1-AS1	Low levels of miR‐133a‐3p and lncRNA RHPN1-AS1 predict poor prognosis.	(264, 265)
		lncRNA EGFR‐AS1 circ_0005909	High levels of lncRNA EGFR‐AS1 and circ_0005909 predict poor prognosis.	(266, 267)
CRC	Diagnosis	miR-325 lncRNA MEG3	Low levels of miR-325 and lncRNA MEG3 predict poor response to chemotherapy.	(268, 269)
		miR-454-3p	Upregulated miR-454-3p is related to a poor response to OXA-based treatment.	(101)
	Prognosis	miR-302a lncRNA HAND2-AS1	Low levels of miR-302a and lncRNA HAND2-AS1 predict poor prognosis.	(94, 270)
		lncRNA AGAP2-AS1 circHIPK3	High levels of lncRNA AGAP2-AS1 and circHIPK3 predict poor prognosis.	(271, 272)
BC	Diagnosis	miR-24-3p LINC00160	High levels of miR-24-3p and LINC00160 predict poor response to chemotherapy.	(120, 273)
		lncRNA CBR3-AS1 circWAC	High levels of lncRNA CBR3-AS1 and circWAC predict poor prognosis.	(274, 275)
HCC	Diagnosis	LINC00680 circRNA-SORE	High levels of LINC00680 and circRNA-SORE predict poor response to chemotherapy.	(276, 277)
	Prognosis	circRNA_101237	High serum level of circRNA_101237 is related to a poor survival of patients (P<0.001).	(278)
PC	Diagnosis	miR-20a-5p	MiR-20a-5p level can serve as a predictor of gemcitabine resistance with an AUC of 89% (P<0.0001), for its downregulation correlates with poor response to gemcitabine.	(279)
	Prognosis	microRNA-296-5p lncRNA HCP5	High levels of microRNA-296-5p and lncRNA HCP5 predict poor prognosis.	(280, 281)
Glioma	Prognosis	miR-1246	Overexpression of miR-1246 predict a low OS in high grade glioma patients.	(282)
Multiple Myeloma	Diagnosis	exosomal circMYC	Upregulated expression of circulating exosomal circMYC correlates with decreased sensitivity to bortezomib.	(283)

In a recent study by Xu et al., they found that the plasma levels of ZFAS1, SNHG11, LINC00909 and LINC00654 were significantly downregulated in postoperative CRC patients compared to preoperative CRC patients. The combination of these four lncRNAs exhibited high diagnostic performance for CRC (AUC = 0.937), especially early-stage disease (AUC = 0.935). Moreover, SNHG11 exhibited the greatest diagnostic ability to distinguish precancerous lesions from early-stage tumor formation (286). Besides, Meng et al. showed that lncRNA BCAR4 overexpression was closely associated with lymph node metastasis (p < 0.001), high tumor stage (p < 0.001), and distant metastasis (p < 0.001). Cancer patients with upregulated lncRNA BCAR4 exhibited poor OS (p < 0.001), suggesting that lncRNA BCAR4 is a promising prognostic biomarker in cancer patients (287). CircRNAs are also promising biomarker candidates in cancer treatment. Liu et al. revealed that hsa_circRNA_101237 was significantly upregulated in multiple myeloma (MM) cells, bortezomib-resistant MM cells, and the bone marrow tissues of MM patients. The high expression of hsa_circRNA_101237 reduced the sensitivity of the MM patients to bortezomib. Further, the AUC value for hsa_circRNA_101237 was 0.92 (p < 0.0001). MM patients with upregulated hsa_circRNA_101237 also demonstrated shorter OS and progression-free survival (PFS). This data indicated that hsa_circRNA_101237 possessed great potential as a diagnostic and prognostic biomarker for MM (288). Collectively, these studies strongly suggest that ncRNAs are valuable biomarkers for diagnosis, prognosis, and predicting drug response in cancer treatment. However, larger patient cohorts are required to further validate their potential as biomarkers in clinical applications.

### Therapeutic potential of ncRNAs in cancer drug resistance

The poor response of patients to chemotherapy and the emergence of drug resistance are still the most critical obstacles in clinical cancer treatment. A large number of studies have confirmed the essential roles of ncRNAs in the development of cancer drug resistance (289). They may act as oncogenes or tumor suppressors to play dual roles in cancer progression, depending on their diverse downstream targets (290). These characteristics endow ncRNAs with great potential as promising therapeutic targets or therapeutic agents in cancer treatment. Therapeutic strategies that make use of ncRNAs or directly target ncRNAs may bring great benefits to the precise treatment of cancer patients, particularly those demonstrating a poor response to chemotherapy. The delivery of tumor-suppressive ncRNAs to target cancer cells is considered a promising strategy to improve cancer intervention. For instance, miRNA-3662 was found to be downregulated in pancreatic ductal adenocarcinoma (PDAC) tissues and cell lines. MiRNA-3662 overexpression enhanced gemcitabine sensitivity and inhibited aerobic glycolysis in the PDAC cells by decreasing hypoxia-inducible factor 1α (HIF-1α) expression (126). LncRNA ENSG0000254615 was found to be highly expressed in 5-FU-sensitive CRC cells. ENSG0000254615 overexpression inhibited cell proliferation and 5-FU resistance by upregulating p21 and downregulating Cyclin D1 in CRC (291). Circ‐G004213 was significantly upregulated in CDDP-sensitive liver cancer cells and its high expression was positively associated with the prognosis of patients with liver cancer. Further analysis revealed that circ‐G004213 suppressed CDDP resistance by upregulating PRPF39 *via* sponging miR‐513b‐5p (292). Therefore, the upregulation of tumor-suppressive ncRNAs, such as miRNA-3662, ENSG0000254615, and circ‐G004213, may represent an effective way to inhibit cancer progression and reverse drug resistance. Targeting oncogenic ncRNAs could be another effective strategy to overcome cancer drug resistance. For instance, miR-192 was significantly increased in CDDP-resistant lung cancer cells compared to non-resistant cancer cells. The overexpression of miR-192 activated the NF-κB signaling pathway by directly targeting NF-κB repressing factor, resulting in the inhibition of apoptosis, promotion of proliferation, and enhancement of CDDP resistance in the lung cancer cells. MiR-192 knockdown obtained the opposite effect (293). In another study, circFBXL5 was found to be highly expressed in BC tissues and 5-FU-resistant BC cells. CircFBXL5 knockdown enhanced the 5-FU sensitivity in the BC cells by suppressing cell migration and invasion and facilitating apoptosis. Mechanistically, circFBXL5 promoted 5-FU resistance by upregulating HMGA2 *via* sequestrating miR-216b (294). Oncogenic ncRNAs, such as miRNA-3662 and circFBXL5, might be used as ideal candidates for therapeutic targets. These findings strongly suggest that the activation of tumor-suppressive ncRNAs or the inactivation of oncogenic ncRNAs are critical mechanisms that restore cancer drug sensitivity. A better understanding of the molecular mechanism of ncRNAs involved in cancer progression and drug resistance will substantially contribute to the precise treatment of cancer patients. However, there are still some challenges that need to be addressed, such as, low bioavailability, side effects, and off-target effects.

## Conclusion and perspective

**Figure 3:**
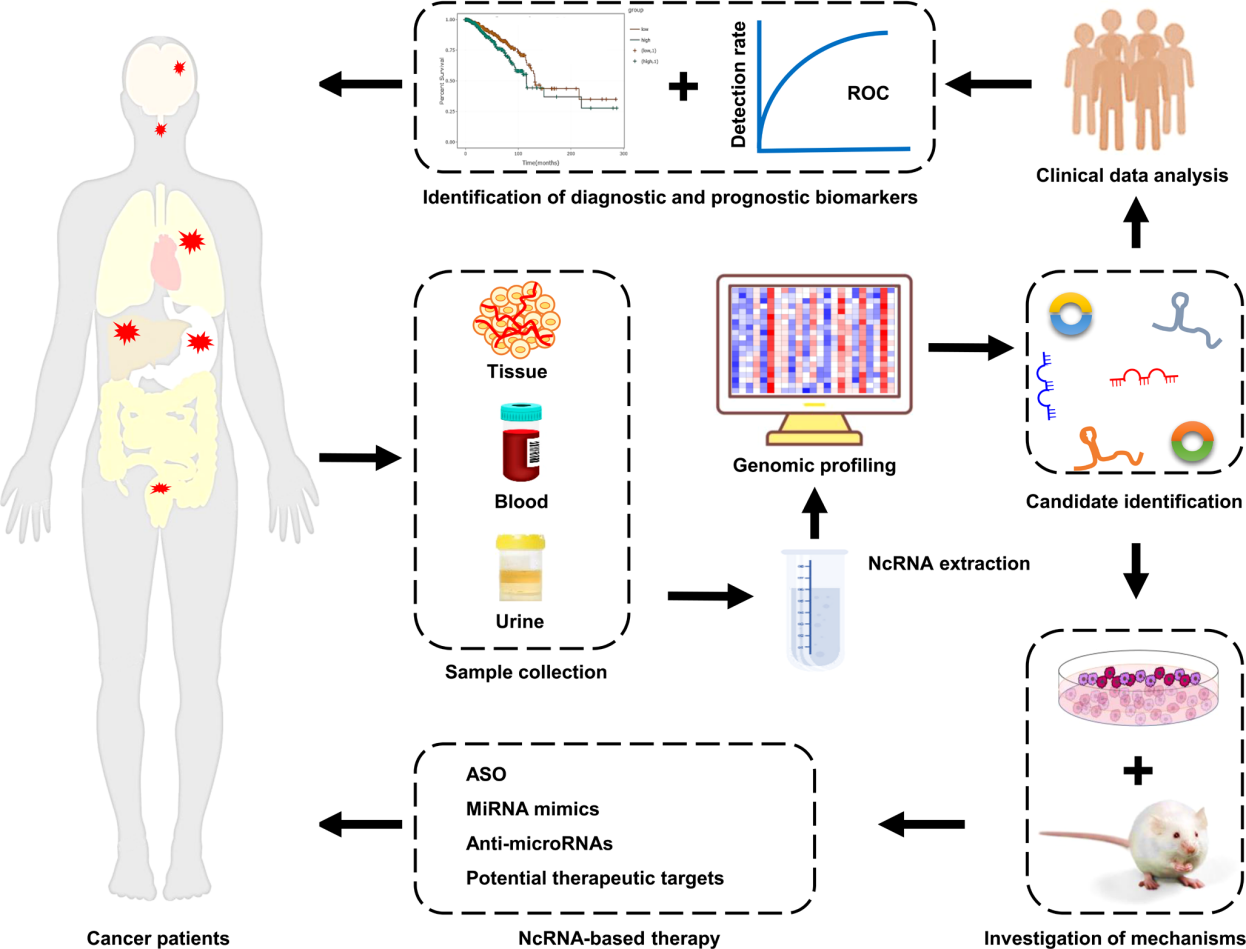
Clinical implications of ncRNAs in cancer drug resistance. NcRNAs are enriched in tissue, blood, and urine samples from cancer patients with drug resistance. The expression profiles of ncRNAs are mapped using high-throughput sequencing technologies. Next, the differentially expressed ncRNAs are screened and identified by bioinformatics analysis. Subsequently, the mechanisms of ncRNAs in cancer drug resistance are elucidated using cell and animal models. The aberrantly expressed ncRNAs that possessed great potential as biomarkers and/or therapeutic targets are identified. Finally, cancer patients, particularly those with drug resistance, receive the individualized precision treatment strategies.

Cancer is one of the most common and fatal malignant diseases worldwide, with high rates of metastasis and recurrence. Chemotherapy remains the best choice for all stages of cancer, and it can effectively improve patients’ prognosis. However, the emergency of drug resistance seriously restricts the clinical efficiency of chemotherapy, and ultimately results in treatment failure. Therefore, a better understanding of the mechanisms responsible for cancer drug resistance will be of great benefit to the development of precise therapeutic strategies for cancer patients, particularly those demonstrating a poor response to chemotherapy. With the rapid development of high-throughput sequencing techniques, a large number of ncRNAs, particularly miRNAs, lncRNAs, and circRNAs, have been found to be aberrantly expressed in cancer tissues and cell lines. These aberrantly expressed ncRNAs are closely associated with cancer progression and drug resistance. It is well established that ncRNAs participate in the development of cancer drug resistance *via* distinct mechanisms, including the suppression of cell death pathways, induction of excessive drug efflux, facilitation of autophagy, regulation of CSC features, and enhancement of the EMT. Aberrant levels of ncRNAs have been observed in cancer patients’ blood, tissue, and even urine (295). Furthermore, the aberrant expression of ncRNAs was found to be closely associated with some pathological characteristics of cancer patients, including OS and PFS (288). These features endow ncRNAs with great potential as ideal biomarkers for the diagnosis and prognosis of cancer patients. In addition, due to the crucial roles of ncRNAs in cancer progression and drug resistance, they are considered to be promising therapeutic targets or therapeutic agents for cancer patients (**Figure 3**). The direct delivery of tumor-suppressive ncRNAs to target cancer cells is a promising way to improve cancer intervention. On the other hand, silencing oncogenic ncRNAs is also an effective strategy to overcome cancer drug resistance. Therefore, it is urgent to develop efficient and non-toxic delivery systems and ncRNA silencing technologies. Although some progress has been made in this area, overcoming resistance to chemotherapeutic drugs remains a large challenge. More clinical trials need to be launched to advance the development of ncRNA-based therapeutic strategies to benefit cancer patients.

## Author contributions

XZ: original draft preparation and writing—review and editing. XA: data curation and funding acquisition. ZJ: data curation. YiwL: data curation. SK: data curation. CD: data curation. JZ: data curation. JW: data curation. YinL: writing—conceptualization, original draft preparation, and writing—review and editing. All authors contributed to the article and approved the submitted version.

## Funding

All authors are supported by Qingdao Medical College, Qingdao University. This work was funded by the China Postdoctoral Science Foundation (2018M642607).

## Conflict of interest

The authors declare that the research was conducted in the absence of any commercial or financial relationships that could be construed as a potential conflict of interest.

## Publisher’s note

All claims expressed in this article are solely those of the authors and do not necessarily represent those of their affiliated organizations, or those of the publisher, the editors and the reviewers. Any product that may be evaluated in this article, or claim that may be made by its manufacturer, is not guaranteed or endorsed by the publisher.
